# Supplementary material for: Motivations for sustained use of modern contraceptives in a peri-urban area: An analytical cross-sectional study
Source: PLoS One. 2025 Jul 14;20(7):e0323434. doi: 10.1371/journal.pone.0323434 (PMC12258606; doi:10.1371/journal.pone.0323434)
Supplement: Attachment — Submitted filename: RESPONSE LETTER, SUSTAINED CONTRACEPTIVE USE.docx [file pone.0323434.s002.docx]

PONE-D-24-29949

MOTIVATIONS FOR SUSTAINED USE OF MODERN CONTRACEPTIVES IN A PERI-URBAN AREA: AN ANALYTICAL CROSS-SECTIONAL STUDY.

PLOS ONE

3/01/25

RESPONSE TO REVIEWERS

Dear Sirs/Madam,

RESPONSE TO REVIEWERS

Thank you for responding to the manuscript. I am grateful for your review and I hope to meet all the requirements.

Thanks.

REVIEWER RESPONSE

1. The abstract could be more concise. Aim to streamline sentences to focus on key findings without losing essential details. RESPONSE: Changes have been made please.
2. Clearly state the primary objective of the study at the beginning of the abstract. Instead of stating “this study sought to determine,” use “this study aimed to determine.” RESPONSE: Changes have been effected.
3. Briefly mention the specific type of questionnaire used and any relevant details about its validity or reliability. This adds credibility to the methodology. RESPONSE: Researchers used open ended and closed questionnaire with face-to-face interview.
4. When presenting results, consider specifying the significance level (e.g., p-values) for the odds ratios (aORs) mentioned. This provides context for the strength of the associations. RESPONSE: changes effected.
5. Strengthen the conclusion by emphasizing the practical implications of the findings for policymakers or health practitioners, perhaps by suggesting specific interventions. RESPONSE: Changes have been made to the conclusion please.
6. Provide more detailed statistical analysis in the results. For example, include confidence intervals for the odds ratios to convey the precision of estimates. RESPONSE: Changes effected please.
7. Expand on the socio-demographic section by providing a clearer picture of the sample. Consider including additional relevant variables (e.g., age distribution, income level) that might influence contraceptive use. RESPONSE: Thank you for drawing our attention to these other variables. Unfortunately, our initial data collection and analysis did not include these variables.
8. When discussing the current use of contraceptives, consider including reasons for choosing specific methods in a summarized format. This could be a table outlining key reasons for method choice. RESPONSE: Thanks for the input. The study however did not look into reasons why certain methods are preferred by our clients.
9. Ensure that terms like "sustained users" are defined clearly when first introduced. This will help readers unfamiliar with the study context. RESPONSE: Well noted.
10. Consider conducting and reporting subgroup analyses based on socio-demographic factors (e.g., age, education level) to understand how motivations for sustained use may differ across groups. RESPONSE: Thank you. We tried estimating the parameters for each of the age groups. Because each group (independent variable) was highly correlated, the estimates were statistically insignificant. They had p value of 1.000. as a result, we decided to treat age as a continuous variable to address the issue of multicollinearity.
11. Expand on the socio-economic motivations for sustained use. Include qualitative data or direct quotes from participants that highlight the diversity of motivations beyond statistics. RESPONSE: Thanks for drawing our attention. The study did not consider qualitative variables that might have an influence on sustained use.
12. While the results provide insight into motivations, consider briefly discussing the challenges respondents faced with their methods, as this could enrich the context. RESPONSE: Challenges respondents faced has been included. Line number 502)
13. Acknowledge any limitations directly in the results section, especially if there were potential biases or limitations in the sample selection. RESPONSE: has been noted.
14. Suggest areas for future research based on the findings. This could help highlight gaps in knowledge that need to be addressed moving forward. RESPONSE: Noted.
