# Supplementary material for: Motivations for sustained use of modern contraceptives in a peri-urban area: An analytical cross-sectional study
Source: PLoS One. 2025 Jul 14;20(7):e0323434. doi: 10.1371/journal.pone.0323434 (PMC12258606; doi:10.1371/journal.pone.0323434)
Supplement: Attachment — Submitted filename: RESPONSE TO REVIEWERS.docx [file pone.0323434.s003.docx]

**RESPONSE TO REVIEWERS**

**MOTIVATIONS FOR SUSTAINED CONTRACEPTIVE USE**

**7/5/25**

Dear Editors,

Thank you for the recommendations and review questions.

RESPONSE TO REVIEWER

1. What is sustainability? As you have said, sustained. Do you think there is sustainability or continuity in using contraceptives?

*RESPONSE: Sustained use is usage of a particular modern contraceptive by a client for 2 or more years.*

2. - Where is the operational definition section also defining motivation, sustained, etc?

*RESPONSE: Operational definition added to the methodology section; page 4.*

3. Measuring sustainability with a cross-sectional study is not proper enough. Have you matched the record from the health facility with the response from women? The authors should clarify this

*RESPONSE: We used both hospital records and response from participants for data gathering. Anomalies between hospital records and interview information were noted and excluded from the data. We have recommended a more advanced study in the conclusion to further determine sustainability of contraceptive use. Thank you.*

4. The result section doesn't clearly show what sustained users mean. Where is the operational definition? Who are new users?

*RESPONSE: Sustained users has been defined on page 11, line 262 to 263 at the results section. I have highlighted it to be easily identified.*

*New users have been included in the operational definitions on page 4; line 102-103. Thank you.*

5. Education and economy are the most important well-known factors, which are not new findings of this study. What are the unique findings specific to this manuscript?

*RESPONSE: Clients who experienced privacy in their counselling sessions for contraceptive uptake were more likely to have sustained use of a particular contraceptive method. Providing privacy and confidentiality is an important element in contraceptive counselling. However, this is not always available in resource constraint environments like the settings of this study. Clients who are limiters compared to spacers are also more likely to have sustained use of contraception as is a finding in this study. This can be found at the result section on page 20, line 402-405. Thank you.*
